# The Ocular Manifestations of Individuals With Down Syndrome: A Systematic Review and Meta-Analysis

**DOI:** 10.1155/joph/2317959

**Published:** 2025-03-18

**Authors:** Jessica A. Beresford-Webb, Emily Charlesworth, Shahina Pardhan, Valerie Wang, Megan Vaughan, Mary Igbineweka, Shahid H. Zaman

**Affiliations:** ^1^Department of Psychiatry, University of Cambridge, Cambridge, UK; ^2^Vision and Eye Research Institute (VERI), Anglia Ruskin University, Cambridge, UK

## Abstract

**Background:** Down syndrome (DS) is the most common genetic cause of intellectual disability. Ocular manifestations occur frequently in people with DS (pwDS) but to date, there is no systematic review or meta-analysis of these conditions across the lifespan.

**Methods:** PubMed, Medline, Embase, Web of Science and Scopus were searched for observational studies reporting ocular manifestations in pwDS, without limiting publication date. The proportion of pwDS with specific ocular manifestations were meta-analysed to obtain a pooled incidence using a random effects model. Sources of heterogeneity were assessed using a meta-regression analysis. For manifestations reported, but without sufficient prevalence data available, a narrative approach was adopted.

**Results:** The search identified 1208 papers. Reviewers independently screened the abstracts, and 54 studies were found to fit the criteria. The age range of the individuals was birth to 88.7 years. Ocular manifestations from highest to lowest prevalence included refractive errors (69.97%, 95% CI 59.95%–79.13%), strabismus (31.41%, 95% CI 24.66%–38.57%), lens opacities (13.79%, 95% CI 8.61%–19.86%), nystagmus (12.72%, 95% CI 9.02%–16.92%) and keratoconus (9.34%, 95% CI 2.47%–19.26%). Alterations of lens and corneal morphology, posterior segment anomalies (including glaucoma) and Brushfield spots were also identified.

**Conclusions:** The ocular manifestations of pwDS are common but varied. Age and/or ethnicity may influence the prevalence of certain ocular manifestations. The level of intellectual disability may also affect the prevalence of ocular manifestations as the prevalence of ocular disorders is known to increase with the severity of intellectual disability in pwDS.

## 1. Introduction

Down syndrome (DS) is the most common genetic developmental disorder, affecting approximately one in 1000 live births worldwide [1]. Ninety-five percent of DS cases are caused by full trisomy 21, around 5% are due to translocation and approximately 2% are mosaic [2]. People with DS (pwDS) display various phenotypes that affect multiple organ systems. These include cognitive defects, premature Alzheimer's disease (AD), hypothyroidism, congenital heart defects, hearing defects and visual problems [2].

Ocular manifestations are common and varied in DS, some of which result in significant visual impairment. Previous studies have found that the number and severity of ocular disorders increase with age [3, 4]. This is of particular importance as the life expectancy of adults with DS has increased substantially [5]. The level of intellectual disability has shown to impact the prevalence of ocular manifestations. The prevalence of ophthalmic manifestations is shown to increase significantly with the level of intellectual disability [3, 6].

Ophthalmological complications are a recognised feature of DS, but no meta-analysis of the published research regarding ophthalmological conditions across the lifespan of pwDS has been conducted. Reliable estimates of the prevalence and epidemiological characteristics of ocular manifestations in pwDS are essential for understanding the mechanisms underpinning these conditions and developing effective prevention and/or treatment strategies for disorders that can significantly impair the quality of life for pwDS. The purpose of this systematic review and meta-analysis is to describe prevalence rates for the most frequently reported ocular manifestations in pwDS and, where appropriate, to discuss the epidemiology, presentation and mechanisms of these manifestations. Definitions of the ophthalmological conditions associates with DS are shown in Appendix 1.

## 2. Methods

### 2.1. Protocol and Registration

This systematic review and meta-analysis followed the Preferred Reporting Items for Systematic Reviews and Meta-Analyses (PRISMA) [7] guidelines and was registered in the PROSPERO database (ID CRD42021237449).

### 2.2. Selection Criteria

The population (P), exposure (E), comparator (C), outcome (O) (PECO) [8] acronym was applied as follows: pwDS (P); clinical or genetic diagnosis of DS (E); pwDS without ocular manifestations and where applicable, people with ocular manifestations without DS (C); description of ocular manifestations in pwDS (O). Inclusion criteria were primary observational studies including pwDS, published in the English language. Interventional studies, grey literature, studies including data reported in another published study and other types of publications were excluded.

#### 2.2.1. Inclusion Criteria

The inclusion criteria include the following:

• Observational studies
• Individuals of any age with a clinical or genetic diagnosis of DS with ocular manifestation data collected
• Descriptions of the ocular manifestations provided
• Printed in English

#### 2.2.2. Exclusion Criteria

The exclusion criteria include the following:

• Studies found in grey literature
• Studies including data reported in another published study

### 2.3. Search Strategy

A literature search was conducted in February 2021 without limiting publication date in PubMed, Medline, Embase, Web of Science and Scopus. The same search strategy was conducted in October 2023, including publications from 2021 to 2023. Medical Subject Headings (MeSH) terms were used and included “Down syndrome” combined (using Boolean operator “AND”) with “vision, low”; “eye abnormalities”; “vision, ocular”; “eye”; “retina”; “amblyopia”; “orbit” (see Table 1). A manual check of the reference lists was conducted.

### 2.4. Study Selection

**Figure 1:**
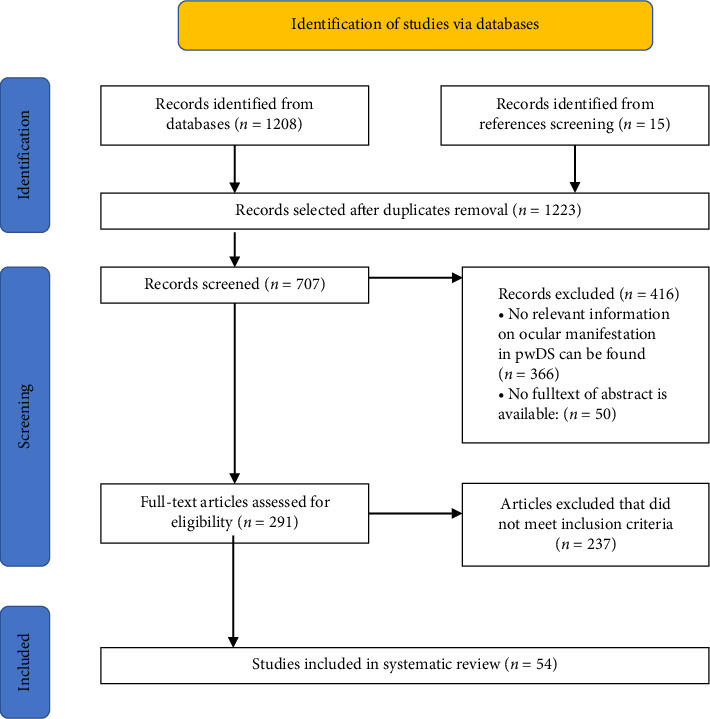
PRISMA flowchart showing the number of papers at each stage of the review process.

The selection of articles was made in three stages (Figure 1). Two researchers (JBW and MI) independently reviewed the titles and abstracts of the studies based on the inclusion and exclusion criteria during the initial search. The additional search was conducted independently by EC and MV. Disagreements were resolved by consensus with MV. JBW and MI independently reviewed 50% of the potentially eligible studies in full to ensure inclusion criteria was met. MV checked 10% of each author's review, and any discrepancies were discussed, and consensus reached. The reference list and citation chains were screened for each paper included. All papers were stored in Mendeley and checked for duplicates. A PRISMA flow diagram was used to document each step (see Figure 1).

### 2.5. Quality Assessment

The methodological quality of all articles meeting the inclusion criteria was assessed according to the methodological design using the National Heart, Lung and Blood Institute of the National Institutes of Health (NIH) tool for quality assessment of observational cohort and cross-sectional studies and case-control studies by two authors (JBW and VW). The NIH tool is a tailored quality assessment tool to assist reviewers in identifying flaws in study methods, implementation and internal validity. The tool includes 14 criteria that each study is assessed against and provides an overall rating of good, fair and poor. Those with a poor-quality rating were excluded from this review [9].

### 2.6. Data Extraction

The variables extracted from each study included the author, location, study design, number and age of participants included; the prevalence and description of ocular manifestations reported and a clinical or genetic diagnosis of DS. Data extraction was completed by JBW, VW, EC and MV; discrepancies were resolved by discussion.

### 2.7. Statistical Analysis

Meta-analysis summarised prevalence estimates for ocular manifestations among pwDS. Pooled estimates of proportions with corresponding 95% confidence intervals (95% CI) were calculated using the Freeman–Tukey double arcsine transformation due to its ability to address issues regarding variance from extreme proportions (less than 0.2 or greater than 0.8) [10, 11]. The *I*^2^ statistic estimated heterogeneity and risk of bias, specifically publication bias, was based on contour-enhanced funnel plots, trim and fill plots, Begg's test and Egger's test [12]. *I*^2^ values of 50% or more indicated substantial heterogeneity. Due to differences in the definitions of specific ocular manifestations, methods of evaluation, study locations, participant age groups and sample sizes across the included studies, a random-effects model was used. Potential sources of heterogeneity were investigated using subgroup and meta-regression analyses, including location (Europe, North America, East Asia or rest of the world), sample size (over 100 or below 100) and age of cohort (< 20 years, ≥ 20 years or < 20 years and ≥ 20 years). These age groups were chosen due to the distribution of ages across the studies included in the meta-analysis. Older age groups could not be assessed accurately due to the lower number of participants in these groups. Sensitivity analyses were performed by omitting one study at a time and calculating a pooled estimate for the remaining studies. Statistical analyses were performed using R version 4.1.2 (R Foundation for Statistical Computing, Vienna, Austria) using the packages referenced in Table S1 [13–22]. Statistical significance was defined as *p* < 0.05.

### 2.8. Synthesis of Results

Ocular manifestations where prevalence data were reported in a minimum of 10 studies were pooled and analysed [23]. Manifestations reported in the studies but without sufficient prevalence data available were not included within the meta-analysis. These reports are synthesised and discussed below.

## 3. Results

### 3.1. Study Selection

One thousand and eight articles were identified. After excluding duplicates, the titles and abstracts of 707 articles were screened, and the full text of 291 articles were assessed for eligibility. Fifty-four studies are included in this systematic review (Figure 1).

### 3.2. Summary of Included Studies and Risk of Bias

Table 2 summarises the 54 studies included in this systematic review. Two studies were conducted across two locations (Spain and Egypt, Macedonia and Croatia) [24, 25]. Of the remaining 52 studies, three were conducted in Africa [26–28], eight in Western Asia [29–36], three in south Asia [37–39], five in East Asia [40–44], four in South America [45–48], 16 in Europe [4, 49–63] and 13 in North America [64–76]. Fourteen studies included a clinical diagnosis of DS, eight a clinical or genetic diagnosis and 32 included a genetic diagnosis. Twenty studies reported the karyotype for participants; three of these failed to report data for all included participants. Age of participants ranged from birth to 88 years. Sample size ranged from one to 1207. All the 54 studies met the necessary quality criteria; 34 classified as good and 20 as fair.

### 3.3. Prevalence of Ocular Manifestations

**Figure 2:**
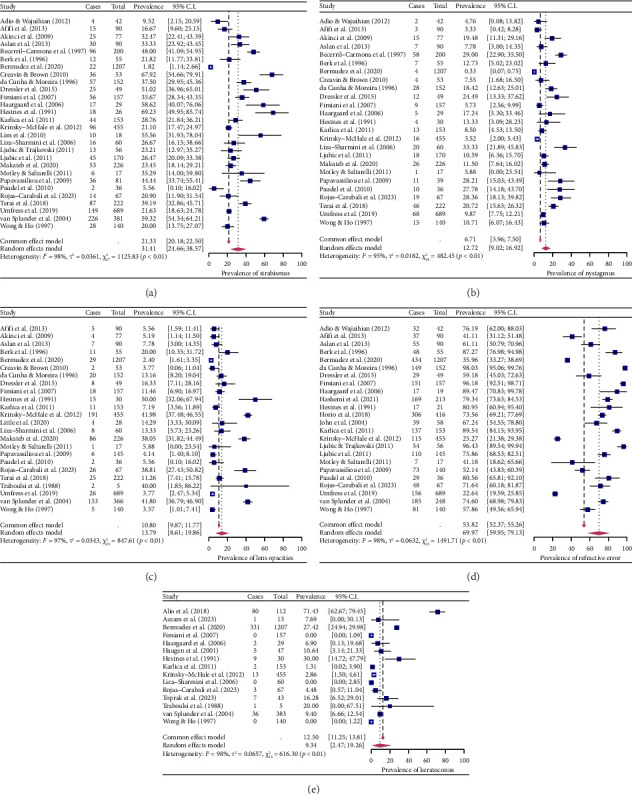
Forest plots for the prevalence of (a) strabismus, (b) nystagmus, (c) lens opacities, (d) refractive error and (e) keratoconus in pwDS.

**Figure 3:**
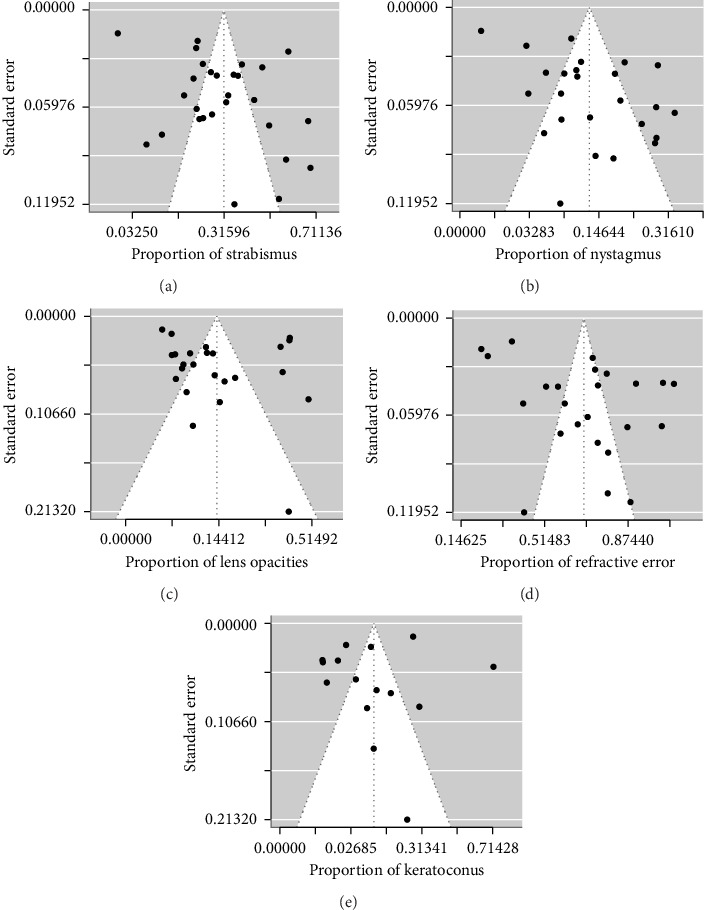
Funnel plots. Each point represents a separate study for (a) strabismus, (b) nystagmus, (c) lens opacities, (d) refractive error and (e) keratoconus. The vertical line represents the mean effect size. The points are distributed asymmetrically, indicating the existence of publication bias (a–e). However, the trim-and-fill plots identified potential missing studies only for nystagmus and refractive error (see Figure S1). Egger's and Begg's tests indicated insignificant bias for all ocular manifestations (*p* > 0.005, see Table S2).

For each ocular manifestation, all studies included in this meta-analysis reported the complete number of cases and the total sample size. Strabismus was reported in 28 studies with a pooled proportion of 31.41% (95% CI 24.66%–38.57%, *I*^2^ = 98%) (Figure 2(a)). Nystagmus was reported in 25 studies with a pooled proportion of 12.72% (95% CI 9.02%–16.92%, *I*^2^ = 95%) (Figure 2(b)). For lens opacities, the pooled prevalence from 24 studies was 13.79% (95% CI 8.61%–19.86%, *I*^2^ = 97%) (Figure 2(c)). The pooled prevalence was 69.97% (95% CI 59.95%–79.13%, *I*^2^ = 98%) for refractive errors from 24 studies and 9.34% (95% CI 2.47%–19.26%, *I*^2^ = 98%) for keratoconus from 15 studies (Figures 2(d) and 2(e)). Substantial heterogeneity was identified across all estimates (*I*^2^ > 50%). Sensitivity analyses revealed that no individual study affected the pooled effect size for all ocular manifestations apart from keratoconus. Alio et al. had a significant influence on the pooled effect size for keratoconus [24]. After removing this study, the pooled proportion for keratoconus was 6.04% (95% CI 1.75%–12.12%). Publication bias was assessed via funnel plots (Figures 3(a), 3(b), 3(c), 3(d) and 3(e)) and trim and fill plots (see Figure S1) followed by Egger's test and Begg's test (*p* > 0.05, for all ocular manifestations, see Table S2), with results indicating an insignificant level of publication bias.

### 3.4. Sources of Heterogeneity: Meta-Regression

Three covariates were examined as potential sources of heterogeneity (age, location and sample size). Sample size was not significantly associated with the prevalence of any ocular manifestation assessed in this review (*p*=>0.05). Significant estimates were found for the covariates of age and location. The *R*^2^ (amount of heterogeneity accounted for) and *P* values for each covariate were as follows: *R*_age_^2^=40.85%, *P*_age_ < 0.01 for lens opacities; *R*_location_^2^=13.91%, *P*_location_=0.04 for strabismus; R_age_^2^=55.97%; *P*_age_ < 0.01 and *R*_location_^2^=29.08%; *P*_location_=0.03 for keratoconus. A subsequent multivariate mixed-effects meta-regression model was developed for keratoconus based on age and location. These two covariates significantly accounted for 51.83% of the heterogeneity (*P*_age+location_ < 0.01).

### 3.5. Variations in Prevalence of Ocular Manifestations

**Figure 4:**
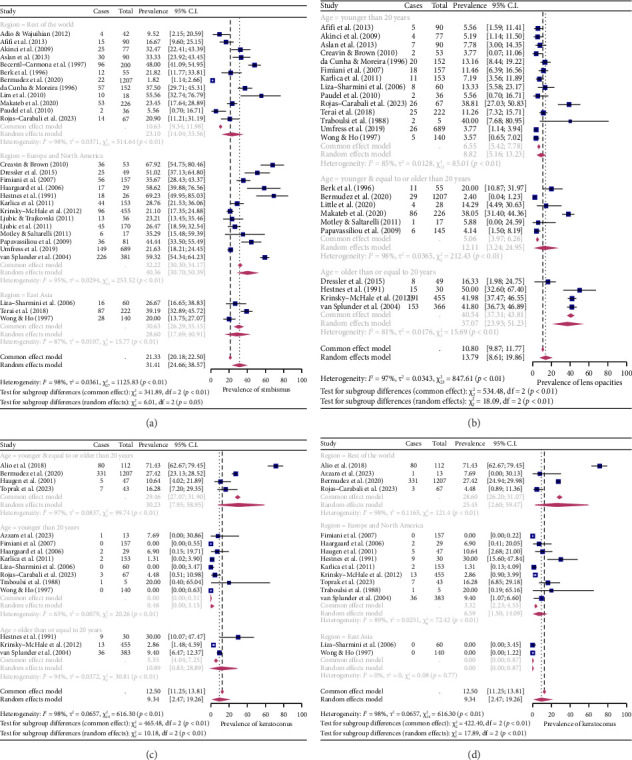
Forest plots for the subgroup analysis of ocular manifestations in pwDS. (a) Forest plot for the prevalence of strabismus by location, (b) forest plot for the prevalence of lens opacities by age subgroup, (c) forest plot for the prevalence of keratoconus by age subgroup and (d) forest plot for the prevalence of keratoconus by location subgroup.

Prevalence was analysed by a subgroup according to age and location in the ocular manifestations where these variables had been identified as significant moderators. When the subgroup analysis was performed according to age, the prevalence was higher for lens opacities in the ≥ 20 years group (37.07%, *I*^2^ = 81%) than in the < 20 years group (8.82%, *I*^2^ = 85%) and < 20 years and ≥ 20 years group (12.11%, *I*^2^ = 98%). The prevalence of keratoconus was higher in the < 20 years and ≥ 20 years group (30.23%, *I*^2^ = 97%) than in the ≥ 20 years group (10.89%, *I*^2^ = 94%) and < 20 years group (0.48%, *I*^2^ = 65%). In the location subgroup, the highest prevalence for strabismus was in Europe and North America (40.36%, *I*^2^ = 95%), followed by East Asia (28.60%, *I*^2^ = 87%) and the rest of the world (23.10%, *I*^2^ = 98%). Prevalence of keratoconus was also lowest in East Asia (0%, *I*^2^ = 0), followed by Europe and North America (6.59%, *I*^2^ = 89%) and the rest of the world (25.45%, *I*^2^ = 98%) (Figures 4(a), 4(b), 4(c) and 4(d)).

## 4. Discussion

### 4.1. Strabismus

We reported a prevalence of strabismus in pwDS at 31.41%. The highest prevalence was reported in studies in Europe and North America (40.36%) followed by East Asia (28.60%) and the rest of the world (23.10%), suggesting that ethnicity-based factors may play a role in prevalence of strabismus in pwDS. The prevalence of strabismus has also shown to be higher in children of White ethnicity without DS [77, 78].

Esotropia has been reported as the most common type of strabismus in pwDS (50%–100% of cases) whilst exotropia is far less common (1%–25% of cases) [25, 26, 29–31, 34, 40–43, 46, 47, 52, 56, 57, 59, 60, 62, 67, 71, 74]. Exotropia may be higher in Asian pwDS, although reports are mixed with the incidence varying between adults and children (25% exotropia cases in Japanese children with DS [41] compared to 2.7% in adults with DS from east Asia) [42].

Karyotype may influence the occurrence and aetiology of strabismus in pwDS with the prevalence reported at 46% in people with mosaic DS compared to 39% in those with trisomy-21 [76]. In contrast to the high proportion of esotropia associated with complete trisomy-21, almost equal proportions of esotropia, exotropia and vertical strabismus have been observed in people with mosaic DS [74]. No relationship has been reported between the severity of ID in pwDS and strabismus [4, 57].

In our analysis, the occurrence of strabismus was not associated with age; a finding in line with studies of adults [4, 71] and those aged 10–30 years with DS [34]. However, strabismus may be acquired rather than congenital, occurring more frequently in children with DS aged between 5 and 19 years than in younger participants [47]. A study in Macedonia and Croatia observed acquired esodeviations in 84.4% of esotropic cases with 12.5% owing to congenital esotropia [25]. An earlier study in Mexico found congenital esotropia at a higher prevalence of 49.4% of esotropic pwDS [46]. The disparity in prevalence rates may be due to ethnicity-based factors.

Strabismus has been equally associated with hyperopia, myopia and astigmatism in pwDS [29, 46, 52], although Akinci et al. noted a similar prevalence of hyperopia in pwDS when comparing those with esotropia and those without strabismus [29]. Ljubic et al. concluded that although the presence of strabismus in pwDS may be predictive of a significant refractive error, a refractive error is not necessarily indicative of a strabismus, with their data showing no association between refraction and strabismus in children with DS [25].

### 4.2. Nystagmus

Nystagmus occurs in 12.72% of pwDS in our meta-analysis. The significant heterogeneity observed between studies may be due to the different methods used to assess nystagmus, with only one study employing eye movement recordings (EMRs) [68]. Using EMRs, Weiss et al. used the term ‘gaze holding instability' to describe the heterogeneity of nystagmus and oculomotor changes observed in DS children with infantile nystagmus. Thus, even with EMR data, results may be noisy and do not easily allow for differentiation between nystagmus subtypes in pwDS.

An association between esotropia and nystagmus has been described in multiple studies, with over 60% of nystagmus cases in children with DS being associated with esotropia [25, 46]. Refractive errors have also been associated with nystagmus in pwDS with as many as 94.4% of nystagmus cases associated with significant refractive errors [25, 40].

The relationship between nystagmus and karyotype is unclear with some reports indicating a relatively low prevalence of nystagmus in those with mosaic DS compared to those with full trisomy 21 [74, 76], whilst others show no correlation between karyotype and prevalence of nystagmus [46]. Sample sizes of those with mosaic DS are generally small which may account for the contradictory results observed.

Contrary to reports of distinct prevalence rates of nystagmus in different ethnicities [34, 40], our meta-analysis did not identify location as a significant moderator. Similarly, whilst Makateb et al. showed that the prevalence of nystagmus significantly increased with age (10.4% for those under 20 years and 17% for those over 20 years) [34], our meta-analysis did not reveal any association.

Mechanisms explaining the association between DS and nystagmus have been proposed, but a conclusive rationale has not been established. Weiss et al. proposed that cerebellar hypoplasia (a condition common in pwDS) [79] may contribute to an underlying neurological deficit in gaze-holding instabilities [68]. However, in a study testing this hypothesis, there was no significant association between neuroimaging results and nystagmus [80]. Alternatively, pwDS and nystagmus often have significant refractive errors, suggesting nystagmus in pwDS may be sensory in nature [25, 40]. Sensory nystagmus can also be caused by congenital cataracts, problems with the retina (macula or fovea) or optic nerve (optic nerve hypoplasia), among other things, all of which have been reported in pwDS.

### 4.3. Lens Opacities and Morphology

Our analysis reported a prevalence rate of 13.79% for lens opacities in pwDS. The prevalence was higher in those aged 20 years or older (37.07%) compared to those under 20 years (8.82%) which concurs with other studies that have observed a significant association between the prevalence of lens opacities and age in pwDS [4, 34, 47, 71]. Cataracts in pwDS may largely be of adolescent or presenile onset with a higher prevalence of cataracts being observed in those aged 12 years and older [47] and reports indicating that the average age of onset of cataracts in pwDS is 48.43 years [71]. Indeed, congenital cataracts are reported less frequently (0.44%–37.8%) [29, 34, 42, 43, 45, 47, 71] than age-related cataracts (11.18%–44.9%) [42, 47, 71] in pwDS.

The heterogeneity observed in our analysis may be influenced by the different definitions employed for cataracts and/or lens opacities. Some definitions included clinically significant cataracts whilst others reported on any observable opacity which may result in overlooking optically insignificant opacities in pwDS. Little et al. reported lens opacities in 54% of DS eyes, but only 14% had clinically significant cataracts [51]. Many studies in this review included a population of pwDS registered at hospitals and/or eye clinics which may further exclude the detection of optically insignificant opacities. The limited use of pupil dilation in examinations may also make it difficult to detect smaller lens opacities in pwDS [51]. Thus, the prevalence rates reported are likely to be an underestimation.

A slightly lower prevalence of cataracts has been reported in those with mosaic DS 2.2% compared to those with trisomy 21 (2.2% and 7.7%, respectively) [76].

Cerulean blue-dot cataracts have frequently been reported in pwDS with 50%–94% of cataracts in pwDS being described as blue-dot cataract [34, 42, 51]. The immunohistochemical analysis has suggested that the supranuclear blue-dot cataracts seen in postmortem lenses of pwDS may be a result of accumulation of amyloid-β, such as the patterns observed in individuals with AD [73]. However, Little et al. showed in in vivo analysis of 28 pwDS; the location and morphology of the cerulean cataracts observed did not coincide with those lens opacities identified in the postmortem lenses [51]. Moreover, no association was found between the number of lens opacities and age. As suggested by Little et al, findings from the postmortem lenses may be an artefact resulting from the small sample size [51].

A thinner lens in pwDS (3.27 ± 0.29 mm) compared to healthy controls (3.49 ± 0.20 mm) [61] has been reported, and bilateral posterior lenticonus has been identified in a case study of a 2.5-year old with DS [39]. However, the structural aspects of the lens in pwDS remains understudied, and as such little is known about the underlying causes and impact of these observations.

### 4.4. Refractive Errors

A prevalence of 69.97% for refractive errors in pwDS was observed in our analysis. Although no significant impact of age or location was observed regarding the prevalence rate, and high heterogeneity was present. Some studies have showed a higher incidence of the refractive error in older children, with almost double the incidence in those aged over 6 years compared to those over 2 years [40]. Others have showed that in pwDS under 10 years of age, differences among refractive errors across age groups were not statistically significant [27]. Some studies report hyperopia as more prevalent in those aged between 10 and 15 years and older [52], with others suggesting that children with DS tend to be hyperopic at all ages, but that prevalence of astigmatism increases with age, becoming highly prevalent in teenage years [47, 49]. These observations suggest that relatively insensitive age groupings (over or above 20 years) combined with a lack of distinction between types of refractive errors in our analysis may limit the conclusions drawn.

The prevalence of myopia increases in pwDS aged over 20 years [25]. Myopic fundal changes observed in adults with DS may predispose pwDS and myopia to conditions, such as retinal detachment (a condition observed in 0.4%–1.7% of pwDS) [31, 42, 43, 47, 71].

Concurring with the results from our meta-analysis, there is no evidence that refractive errors significantly differ across ethnicities [47], with no significant sex differences (hyperopia 46.7% male, 44.5% female; myopia 28.7% male, 39.1% female) [36], and similar rates of refractive errors being observed in those with mosaic DS (52.2%, myopia or hyperopia) compared to those with trisomy 21 (52%, myopia or hyperopia) [76].

The observation of significant refractive errors in early childhood that persist and increase with age suggests a lack of emmetropization in pwDS, characterised by abnormal axial development (axial length of 22.44 ± 1.42 mm and 23.31 ± 1.01 mm, for pwDS and controls, respectively) [61]. A thinner and steeper cornea observed in pwDS compared to the general population may be causative factors for astigmatism. However, Haugen et al. reported that pwDS had a statistically higher incidence of astigmatism than healthy controls, even after excluding keratoconus patients [61]. Thickness of the ciliary muscle has been proposed as a physiological mechanism for refractive error development in pwDS; however, similar thickness has been observed between pwDS and control participants [64]. High myopia has been associated with cardiac malformations in pwDS aged between 2 months and 18 years, although this has not been corroborated by any further studies [47].

### 4.5. Corneal Morphology

The prevalence of keratoconus in pwDS in this current review was 9.34%. Using sensitive corneal topographic methods, prevalence of keratoconus reached 71% in a recent study of 112 pwDS aged between three months and 60 years. Older studies using less advanced diagnostic tools may underestimate the occurrence of keratoconus in pwDS. The only study in this review to use advanced diagnostic tools was identified in our analysis as an outlier [24].

Ethnicity may play a role in the prevalence of keratoconus in pwDS, as evidenced by the lack of keratoconus reported in studies from East Asia (0%) compared to the studies from Europe and North America (6.59%) and the rest of the world (25.45%). Although Krinsky-McHale [71] did not find any association between keratoconus and age in adults with DS, our analysis revealed a higher prevalence in those over 20 years compared to those under 20 years; an observation conceivably due to the fact children are more likely to be less cooperative during corneal examination, and therefore harder to examine causing early stages of the condition to be missed.

A thinner and steeper cornea has been reported in pwDS compared to controls [24, 32, 35, 53, 54, 61]. Central corneal thickness has been observed to be between 480 and 516 μm in pwDS compared to 538.95 and 555.7 μm in healthy controls. Reduced corneal thickness as well as reduced volume and higher keratometry values in pwDS compared to controls persist even after excluding pwDS who have indications of keratoconus [24, 32, 53, 54, 61].

Genes on Chromosome 21 may affect the collagen structure of the corneal stroma, which could lead to thinning and deformity of the cornea in pwDS [81, 82]. A thinner cornea leaves the cornea particularly vulnerable to atopy and eye rubbing, both of which have been associated with the development of keratoconus in pwDS [61].

### 4.6. Brushfield Spots

Brushfield spots are a well-known finding in pwDS, with prevalence ranging between 0% and 60%. Of note, Berk et al. observed Brushfield spots more frequently in blue- or hazel-coloured irises which point to potential ethnicity-based differences [31]. The lower prevalence rates observed in studies from Italy, Malaysia and China (0%) [40, 43, 59] compared to those from North America (> 50%) [66, 76] may be due to eye colour differences in the demographics studied.

### 4.7. Posterior Segment Anomalies

Findings from this systematic review suggest that glaucoma in pwDS is rare, with the prevalence ranging between 0% and 6.67%. Intraocular pressure, with or without glaucoma, is lower in pwDS compared to controls and those with other forms of ID (although not significantly) [30], and it has been suggested that pwDS has a decreased risk of ocular hypertension [4]. The DS Critical Region 1 (DSCR1) located on Chromosome 21 has recently been identified as a potential new target for the treatment of glaucoma [83].

Traboulsi et al. [66] observed that two out of five DS children with bilateral glaucoma also had retinal detachment, leading to suggestions that pwDS and congenital glaucoma may also be predisposed to retinal detachment, a condition reported in a number of studies included in this review [31, 42, 43, 47, 71].

An increased number of retinal vessels crossing the optic nerve head, appearing in a spoke-like pattern, has been observed in pwDS [31]. Increased vessel branching [74], vessel tortuosity [59] and superfluous blood vessels [50] have been sporadically reported in pwDS and may be a consequence of altered angiogenesis [84].

Whilst optical coherence tomography studies are rare in pwDS, the findings indicate that pwDS have abnormal development of the retina. In children with DS, when compared to healthy controls, a significantly thicker central fovea (281 ± 17 μm vs. 246 ± 21 μm respectively recorded in one study, with another reporting average thickness for pwDS at 264.33 ± 26.0 μm) and shallower foveal pit have been reported [68, 75]. In babies with DS, a thinner macula compared to controls has been observed, with shallow foveal contours and persistence of inner retinal layers also being identified; the findings are not present in healthy controls [37]. Increased retinal foveal and ganglion foveal thickness and reduced choroidal foveal thickness and choroidal thickness in the temporal and inferior quadrants have been observed in pwDS aged 15–47 years compared to controls [58]. Investigations using a trisomic mouse model suggest that genetic influences, specifically a disorder of apoptosis, may play a role in the abnormal retinal development observed in pwDS [85].

Although less frequently described, other posterior segment anomalies including pallor of the optic nerve (typically optic atrophy due to optic nerve fibre layer thinning), pseudopapilloedema (elevation of the optic disc), preretinal haemorrhage [59], retinoblastoma [43], retinal drusen, lattice degeneration, myelinated nerve fibre [42], retinitis pigmentosa, macular degeneration [71] and uveitis [43] have been sporadically reported in pwDS and may represent coincidental findings.

### 4.8. Limitations

A limitation of this study is that in large continental regions such as Africa, south and central America and southern and western Asia, there were insufficient studies to provide representative estimates for the region. As such, these locations were categorised as the ‘Rest of the World' for the purpose of the analyses, which may obscure differences between these regions. The same is true for studies including participants of different ages for which the data available only allowed for a distinction between those younger or older than 20 years. The prevalence of ocular abnormalities will change with age; however, too few studies included older patients with DS so this could not be investigated further. This in particular will affect the overall prevalence of lens opacities (13.79%) which is known to be higher in older adults with DS [86]. The level of intellectual disability may also affect the prevalence of ocular manifestations. While the studies included whether patients had a clinical or genetic confirmation of DS, few studies included details of the level of intellectual disability patients had, therefore this could not be investigated as part of this review. In addition, the heterogeneity of the included studies regarding the location, age of participants and other unknown factors made it challenging to achieve stable meta-analysis results despite the use of a standardised analysis process. The prevalence rates reported here may be an overestimation due to the potential selection bias of some studies which only included participants who were already registered at eye clinics and/or hospitals. Moreover, whilst the absence of missing data in the studies included in the meta-analyses indicates that they are of high quality and follow consistent reporting practices, reducing the risk of bias from imputation or data exclusion, challenges such as selective reporting of results and the nonpublication of unfavourable findings could still impact our results. Finally, because we only included studies that either clinically or genetically confirmed a diagnosis of DS, several studies including pwDS lacking explicit DS diagnostic procedures were excluded from this review.

## 5. Conclusion

DS is a highly prevalent condition worldwide. This review provides the most comprehensive and up to date systematic review and meta-analysis of ophthalmological manifestations in pwDS. Refractive errors were the most common condition with a prevalence of 69.97%, followed by strabismus at 31.14%, lens opacities at 13.79%, nystagmus at 12.72% and keratoconus at 9.34%. Glaucoma and Brushfield spots were also features of the DS eye, occurring in 0%–6.67% and 0%–60% or pwDS, respectively. Age and/or ethnicity may play a role in the prevalence of these manifestations with strabismus occurring more frequently in European and North American populations, a higher prevalence of lens opacities and keratoconus being observed in older pwDS as well as keratoconus rarely being observed in studies from East Asia.

A key strength of this meta-analysis and systematic review is the strict adherence to the PRISMA guidelines for reporting systematic reviews and meta-analysis, using critical assessment of study quality with strict application of the inclusion and exclusion criteria. Heterogeneity was identified and investigated using a meta-regression model and subgroup analyses. Furthermore, this current review includes studies from a variety of world regions (Europe, Asia, Africa and north and south America) spanning a broad research period (1988–2023), ensuring sufficient breadth to encompass the diversity of studies in this research area, whilst being narrow enough to ensure meaningful results. As a result, our study can be referenced by research peers as a starting point for further research into the ophthalmological manifestations of pwDS.

In conclusion, this meta-analysis and systematic review summarises the epidemiological traits of ocular manifestations observed in pwDS. The findings of this review provide a direction for further studies in this area and will be useful for the design of ophthalmological screening and related public health strategies. Future directions should be mindful of the challenges pwDS face which may limit their insight into and reporting of ocular changes and disturbances.

## Figures and Tables

**Table 1:** Combination of search terms during the initial database searches.

Search terms		
“Down syndrome”	AND	“Vision, low”
		“Eye abnormalities”
		“Vision, ocular”
		“Eye”
		“Retina”
		“Amblyopia”
		“Orbit”

**Table 2:** The 54 studies included in the systematic review.

Authors	Year	Location	DS diagnosis	Sample size	Karyotype	Mean (SD) age	Age range	Quality assessment
Adio and Wajuihian	2012	Nigeria	C	42	NS	11.43 (6.04) years	6–28 years	Fair

Afifi et al.	2013	Egypt	G	90	NS	2.2 (NS) years	3 months–10 years	Good

Akinci et al.	2009	Turkey	G or C	77	NS	8.5 (3.7) years	1–17 years	Good

Al-Bagdady et al.	2010	UK	G or C	182	NS	NS	6 months–15 years	Fair

Alio et al.	2018	Spain and Egypt	G	112	NS	14.88 (15.76) years	NS	Fair

Anderson et al.	2022	USA	C	26	NS	29 (9) years	18–52 years	Good

Asgari et al.	2021	Iran	G	203	NS	17.0 (4.7) years	NS	Good

Asgari et al.	2020	Iran	G	234	NS	With keratoconus: 16.71 (4.25) years Without keratoconus: 17.19 (4.84) years	NS	Good

Aslan et al.	2013	Turkey	G or C	90	NS	8.13 (4.1) years	4–12 years	Fair

Azzam et al.	2023	Egypt	G	13	NS	11.69 (3.71) years	8–18 years	Fair

Becerril-Carmona et al.	1997	Mexico	G	200	Trisomy: 180 Translocation: 16 Mosaicism: 4	9.9 (NS) years	1–40 years	Fair

Berk et al.	1996	Turkey	G	55	Trisomy: 54 Translocation: 1	86 (NS) months	2 months–25 years	Good

Bermudez et al.	2020	Brazil	G	1207	Trisomy: 1142 Translocation: 37 Mosaicism: 28	NS	0–42 years	Fair

Costa	2011	USA	G	32	Trisomy: 30 Translocation: 2	Females: 21.13 (10.86) years Males: 22.31 (7.58) years	14–37 years	Good

Costa	2011	USA	G	32	Trisomy: 30 Translocation: 2	Females: 21.13 (10.86) years Males: 22.31 (7.58) years	14–37 years	Good

Creavin and Brown	2010	England	C	53	NS	NS	0–16 years	Fair

da Cunha and Moreira	1996	Brazil	G	152	Trisomy: 121 Translocation: 2 Mosaicism: 3 Missing data: 26	NS	2 months−18 years	Good

Dressler et al.	2015	Italy	G or C	49	Trisomy: 38 Mosaicism: 8 Unknown: 3	28.7 (8.3) years	19–52 years	Good

Esteban et al.	2021	Spain	G	52	NS	26.2 (11.8) years	15–47 years	Good

Fantin et al.	1998	USA	G	1	Trisomy: 1	Case study, 2 months	N/A	Fair

Fimiani et al.	2007	Italy	G	157	Trisomy: 153 Translocation: 1 Mosaicism: 2 Missing data: 1	5.28 (NS) years	1 month–18 years	Fair

Fong et al.	2013	Hong Kong	C	91	NS	38 (6.5) years	30–56 years	Good

Haargaard and Fledelius	2006	Denmark	C	29	NS	NS	Birth—17 years	Fair

Hashemi et al.	2020	Iran	C	202	NS	16.99 (4.70) years	NS	Good

Hashemi et al.	2021	Iran	C	213	NS	17.2 (4.8) years	10–30 years	Good

Haugen et al.	2001	Norway	G	47	Trisomy: 46 Translocation: 1	20 (3.9) years	14–26 years	Good

Hestnes et al.	1991	Norway	G	30	NS	42 (NS) years	21–72 years	Good

Horio et al.	2018	Japan	C	416	NS	6.1 (4.1) years	0–18 years	Good

Jain et al.	2021	India	G	1	Trisomy: 1	Case study, 2.5 years	N/A	Good

John et al.	2004	UK	G	58	Trisomy: 58	6.66 (NS) years	9 months–12.75 years	Good

Karlica et al.	2011	Croatia	G	153	NS	11.7 (3.2) years	0–18 years	Fair

Krinsky-McHale et al.	2012	USA	G or C	455	Trisomy: 455	50.93 (7.85) years	NS	Good

Lim et al.	2010	Canada	C	18	NS	2 (NS) months	NS	Fair

Little et al.	2020	UK	C	28	NS	24.1 (14.3) years	6–55 years	Good

Liza-Sharmini et al.	2006	Malaysia	G or C	60	NS	6.72 (NS) years	NS	Fair

Ljubic and Trajkovski	2011	Macedonia	G or C	56	NS	14.9 (6.7) years	2–28 years	Fair

Ljubic et al.	2011	Macedonia and Croatia	G or C	170	NS	13.8 (NS) years	1–34 years	Good

Makateb et al.	2020	Iran	G	226	NS	16.05 (4.82) years	10–30 years	Good

Mangalesh et al.	2019	India	G	19	Trisomy: 19	2 (2.1) years	3 months–6.5 years	Good

Moncaster et al.	2010	USA	G	19	NS	NS	1 day–69 years	Fair

Motley and Saltarelli	2011	USA	G	17	Mosaicism: 17	9 (NS) years	6 months–32 years	Good

O'Brien et al.	2015	USA	C	17	NS	11 (3.1) years	6–16 years	Good

Papavassiliou et al.	2009	USA	G	161	Trisomy: 54 Mosaicism: 107	6.6 (NS) years	1 month–43 years	Fair

Paudel et al.	2010	Nepal	C	36	NS	6.94 (5.38) years	NS	Fair

Rojas-Carabali et al.	2023	Colombia	G	67	Trisomy Mosaicism Translocation	12.4 (2.28) years	8–16 years	Good

Terai et al.	2018	Japan	G	222	NS	NS	3 months–20 years	Good

Toprak et al.	2021	Spain	G	43	NS	24.3 (11.3) years	NS	Good

Toprak et al.	2023	Spain	G	43	NS	24.3 (11.3) years	NS	Good

Traboulsi et al.	1988	USA	G	5	Trisomy: 5	NS	6 weeks–20 years	Fair

Umfress et al.	2019	USA	C	689	NS	3.3 (3.8) years	4 weeks–18 years	Good

van Splunder et al.	2004	Netherlands	C	409	NS	45.7 (NS) years	20.2–88.7 years	Good

Vega-Estrada et al.	2020	Spain	G	20	NS	24.03 (11.72) years	NS	Good

Weiss et al.	2016	USA	G	18	Trisomy: 18	4.6 (NS) years	0.4–14.9 years	Good

Wong and Ho	1997	China	G	140	Trisomy: 140	3.74 (NS) years	3 months–13 years	Fair

## Data Availability

The data that support the findings of this study are available from the corresponding author, E.C., upon reasonable request.

## Appendix 1: Definitions of Ophthalmological Conditions

Ophthalmological conditions associated with DS.

  Strabismus: A condition in which the eyes do not point in the same direction, causes one or both eyes to turn; inward (esotropia), outwards (exotropia), upward (hypertropia) and downward (hypotropia) [87].
  Nystagmus: Involuntary rhythmic oscillation of the eyeballs, either pendular or with a slow and fast component [88].
  Lens opacities: A cataract is defined as complete or partial opacity of the ocular lens. Due to the different definitions used for ‘cataracts' across studies included in this current review, we have chosen to use the term, ‘lens opacity' [89].
  Refractive error: The inability to focus light properly on the retina, often corrected by glasses, contact lenses or refractive surgery [90].
  Keratoconus: A conic protrusion of the cornea caused by thinning of the stroma; usually bilateral [91].
  Brushfield spots: Light-coloured condensations of the surface of the mid-iris, seen in DS [92].
  Glaucoma: A disease of the eye characterised by increased intraocular pressure, excavation and atrophy of the optic nerve and produces defects in the field of vision and can lead to eventual blindness [93].

## Supporting Information

The supporting information contains the R packages used in the meta-analysis, publication bias measured by Begg's and Egger's test and trim and fill funnel plots as additional methods for bias assessment.
