# Supplementary material for: The Ocular Manifestations of Individuals With Down Syndrome: A Systematic Review and Meta-Analysis
Source: J Ophthalmol. 2025 Mar 18;2025:2317959. doi: 10.1155/joph/2317959 (PMC11936530; doi:10.1155/joph/2317959)
Supplement: Supporting Information — Additional supporting information can be found online in the Supporting Information section. [file 2317959.f1.docx]

**Supplementary material**

**Table S1.** R packages used in meta-analysis.

**Table S2.** Publication bias measured by Begg’s and Egger’s test.

**†**: Original effect size and 95% confidence interval.

**‡**: Effect size and 95% confidence interval after using trim and fill method.

**Figure S1.** Trim and fill funnel plots. Each filled point represents a separate study for (a) nystagmus and (b) refractive error. Hollow points represent studies identified as missing. The vertical line represents the mean effect size.
